# The Incidence of Congenital Heart Defects in Offspring Among Women With Diabetes in Saudi Arabia

**DOI:** 10.7759/cureus.14225

**Published:** 2021-03-31

**Authors:** Sarah M. M Alyousif, Fisal T Aldokhel, Omar Khalid Alkhanbashi, Majed Hayf A Alqahtani, Abdulrahman Mohammed M Aladawi, Abdullah Abdulrahman Ashmawi, Ada Al-Qunaibet, Emad Masuadi

**Affiliations:** 1 Cardiology, King Saud bin Abdulaziz University for Health Sciences, Riyadh, SAU; 2 Clinical Pharmacist Specialist, Ministry of National Guard Health Affairs Hospital, Riyadh, SAU; 3 Medicine, King Saud bin Abdulaziz University for Health Sciences, College of Medicine, Riyadh, SAU; 4 Epidemiology, Saudi Center for Disease Prevention and Control, Riyadh, SAU; 5 Biostatistics, King Saud bin Abdulaziz University for Health Sciences, Riyadh, SAU

**Keywords:** congenital heart defects, infants, type 1 diabetes mellitus, type 2 diabetes mellitus, gestational diabetes, mothers

## Abstract

Background: The risk of congenital anomalies is increased in infants of diabetic mothers (IDM). The most frequent cardiac anomalies in IDMs include ventricular septal defect, transposition of great arteries, and aortic stenosis.

Objective: Estimating the incidence of infants with congenital heart defects (CHD) whose mothers have diabetes in Saudi Arabia at a tertiary hospital in the National Guard Health Affairs (NGHA) system.

Materials and methods: This study was a retrospective cohort. The population was all births of type 1 and type 2 diabetic mothers and non-diabetic mothers (also mothers with gestational diabetes) in NGHA by following the exclusion criteria, which were mothers over 40 and below 20 years of age, and other risk factors such as drug-induced congenital disease. The data was from deliveries from January 1st 2018 to January 1st 2019. Data were collected by chart review using the Best-Care system at NGHA hospital. Statistical Package for the Social Sciences (SPSS) version 20 (IBM Corp., Armonk, NY, USA) was used for the statistical analysis.

Results: A total of 1838 diabetic mothers and non-exposure, non-diabetic mothers, with the outcome of whether the infant had CHD, were included in this study. Most of the mothers (544, 30.11%) were aged 30-34 years old. About two-thirds of mothers, 1161 (63.24%), weren't diabetic, 500 (27.23%) had gestational diabetes, 132 (7.19%) were type 2 diabetes (T2DM), and 43 (2.34%) were type 1 diabetes (T1DM). Two hundred eighteen (11.82%) offspring had CHD, and the remaining 1625 (88.17%) did not. The most frequent echocardiographic abnormalities in infants of diabetic mothers were patent ductus arteriosus (PDA) (31.75%), patent foramen ovale (PFO) (31.75%), and atrial septal defect (ASD) (23.64%).

Conclusion: The incidence of CHD among infants of included mothers in this cohort study was 11.82%. The most frequent echocardiographic abnormalities in the infants of diabetics were PDA and PFO. The incidence of CHD was higher among mothers who had T1DM followed by T2DM, and whose ages were between 30-34.

## Introduction

Cardiovascular disease (CVD) is the major cause of mortality and morbidity worldwide [1]. Incidence and mortality of CVD have decreased in some countries, but the prevalence of CVD has risen in children and young adults in recent decades [2,3]. The risk factors for CVD change throughout a lifetime and early onset CVD could have a different reason than CVD diagnosed in later adulthood [4]. 

Prenatal susceptibility to maternal diabetes has also been correlated with obesity, congenital heart disease (CHD), and diabetes in offspring. These diseases could lead to an increased risk of CVD in later life [5]. 

Experimental studies suggest that hyperglycemia during early embryogenesis may alter gene expression in key cellular components of the developing heart, particularly the embryonic heart's outflow sections; however, the mechanism producing this altered gene expression is unclear [6,7]. 

In Saudi Arabia, an estimate found that each year 5.4 per 1000 infants suffer from severe CHD [8]. The precise etiology is unknown, but it has multiple genetic and/or environmental factors [9]. For example, increased maternal age, vitamins, folic acid deficiency, phenylketonuria, maternal diabetes mellitus (DM), rubella virus, febrile illness, and influenza are all considered risk factors [10]. Several studies have shown a strong correlation between diabetes in mothers and CHD in infants [4-10]. 

Due to the significant evidence that confirms the strong association between CHD and maternal diabetes, and the lack of studies in Saudi Arabia also the strong impact of CHD on the patient, family, community, and financial effect on the country, this research aims to estimate the incidence of infants with CHD. The mothers of the infants studied had diabetes at a tertiary hospital in the National Guard Health Affairs (NGHA) system in Saudi Arabia.

## Materials and methods

This study was a retrospective cohort because it was based on risk factors and exposure, which were the diabetic mothers, and non-exposure, which were the non-diabetic mothers, and the outcome is whether the infant had CHD or not. It was conducted in tertiary National Guard Hospitals in three Saudi Arabian cities, Riyadh, Jeddah, and Al Hassa. The first NGHA hospital was instituted in May 1983 with a capacity of approximately 3133 beds. Riyadh has 2,101 beds, Jeddah has 751 beds, and Al Hassa has 281 beds. The population was all births of type 1 and type 2 diabetic mothers (T1DM and T2DM), gestational diabetic mothers (GDM), and non-diabetic mothers in NGHA by following the exclusion criteria, which were mothers over 40 and below 20 years of age, and other risk factors such as drug-induced congenital disease. The data is from deliveries from January 1st, 2018 to January 1st, 2019. The estimated number of deliveries during this period was approximately 5,000. Data were collected by chart review using the Best-Care system, an electronic health record, at NGHA hospitals; only the research team members collected the data. Variables were collected from two files, as follows: outcome variable (whether the infant had CHD or not) and grouping variable (whether the mother had diabetes or not).

Regarding the sample size, we assumed that P1=0.05 and P2=0.1 and alpha=0.05 to achieve the power of 80% requiring a sample of 299 diabetic patients and 1205 non-diabetic patients. Non-probability convenient sample by including those who meet the inclusion and exclusion criteria.

Statistical analysis

The program used was Statistical Package for the Social Sciences (SPSS) version 20 (IBM Corp., Armonk, NY, USA). The type of data was descriptive. The categorical data were presented by percentages and frequencies such as gender, CHD, and diabetes. Simultaneously, numerical data was prescribed as mean and standard deviation such as mother's age and baby's age. The relative risk was calculated to evaluate the risk and confidence interval. Logistic regression was carried out to evaluate the risk factors. The test was considered significant if the p-value was less than 0.05.

Ethical considerations

Consent was not required because it was a chart review, all data was kept safe, and no identification data were asked, such as medical record number (MRN), names, and ID (MRN was replaced with serial number). The access to research data was kept only between the group member maintaining the confidentiality and safety of the data, and the collected data was kept safe.

## Results

**Figure 1:**
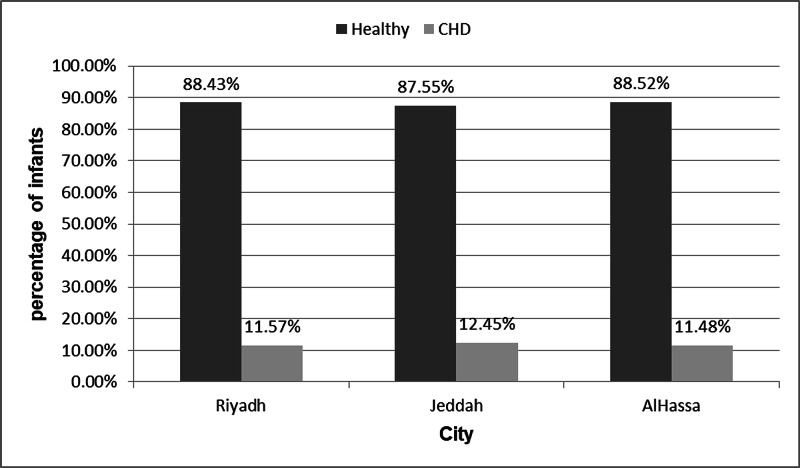
The distribution of included infants based on their cities. CHD - Congenital Heart Defect 1141 is the total births in Riyadh, 132 infants had CHD 466 is the total births in Jeddah, 58 infants had CHD 244 is the total births in AlHassa, 28 infants had CHD

A total of 1838 diabetic mothers and non-exposure, non-diabetic mothers, with the outcome of whether the infant had CHD were included in this study. Table 1 illustrated the demographics of included mothers and their infants. Most of mothers (544; 30.11%) were aged from 30-34 years old, 500 (27.6%) from 35-40 years, 483 (26.73%) from 25-29 years, and 280 (15.50%) from 18-24 years. About two-thirds of mothers (1161; 63.24%) weren't diabetic, 500 (27.23%) had gestational diabetes, 132 (7.19%) were T2DM, and 43 (2.34%) were T1DM. More than half of their offspring (956; 52.02%) were males, while the remaining 882 (47.99%) were females. Regarding the gestational age, the majority of pregnancies (1624; 88.55%) were in term (37-42 weeks), 208 (11.34%) were preterm (<37 weeks) and two (0.11%) were post-term (>42 weeks). Regarding the infants' weight; 1568 (85.78%) were normal weight (2.5-4 kg), 202 (11.05%) were underweight (<2.5 kg) and 58 (3.17%) were overweight (>4 kg). Two hundred eighteen (11.82%) of offspring had CHD, and the remaining 1625 (88.17%) did not. More than half of mothers (1141; 61.64%) were from Riyadh, 466 (25.17%) were from Jeddah, and 244 (13.18%) were from Al Hassa (Figure 1).

**Table 1:** The demographics of included mothers and their infants. T1DM - Type 1 Diabetes Mellitus T2DM - Type 2 Diabetes Mellitus GDM - Gestational Diabetes Mellitus W - Weeks CHD - Congenital Heart Defects

Demographics			Number of participants
Mothers	Age	18-24	280 (15.50%)
		25-29	483 (26.73%)
		30-34	544 (30.11%)
		35-40	500 (27.67%)
	Diabetic status	Non-diabetic	1161 (63.24%)
		TIDM	43 (2.34%)
		T2DM	132 (7.19%)
		GDM	500 (27.23%)
Infants	Gender	Male	956 (52.01%)
		Female	882 (47.99%)
	Gestational age	Preterm (<37w)	208 (11.34%)
		Term (37-42w)	1624 (88.55%)
		Post-term (>42w)	2 (0.11%)
	Weight	Underweight (<2.5kg)	202 (11.05%)
		Normal weight (2.5-4kg)	1568 (85.78%)
		Overweight (>4kg)	58 (3.17%)
	CHD status	Yes	218 (11.82%)
		NO	1625 (88.17%)
	City	Riyadh	1141 (61.64%)
		Jeddah	466 (25.17%)
		Al Hassa	244 (13.18%)

Table 2 shows the CHD incidence among multiple maternal and infant variables, and the association between each type of diabetes in mothers with the incidence of CHD in infants. The higher incidence of CHD among the mothers was reported in the age range from 30 to 34 years old. A higher incidence of CHD was reported among T1DM patients (16; 37.21%). The incidence of CHD among infants was higher among males than females, where it was 116 (12.13%) and 102 (11.56%), respectively. About a quarter of overweight infants (15; 25.86%) had CHD, 31 (15.35%) of underweight, and 172 (10.97%) of normal weight. All of the post-term infants (two; 100%) had CHD, 43 (20.67%) of preterm had CHD, and 173 (10.65%) of in term had CHD. The statistical analysis reported a significant correlation between the diabetic status of mothers, the weight of infants, and gestational age regarding the incidence of CHD (P-values <0.0001, =0.0008, and <0.0001, respectively).

**Table 2:** CHD incidence in each variable. CHD - Chongenital Heart Defect T1DM - Type 1 Diabetes Mellitus T2DM - Type 2 Diabetes Mellitus GDM - Gestational Diabetes Mellitus W - Weeks

Variables			Healthy	CHD
Mothers	Age P-value 0.0694	18-24	246 (87.86%)	34 (12.14%)
		25-29	438 (90.68%)	45 (9.32%)
		30-34	465 (85.48%)	79 (14.52%)
		35-40	445 (89%)	55 (11%)
	Diabetic status P-value <0.0001	Non-diabetic	1042 (89.75%)	119 (10.25%)
		T1DM	27 (62.79%)	16 (37.21%)
		T2DM	98 (74.24%)	34 (25.76%)
		GDM	452 (90.40%)	48 (9.60%)
Infants	Gender P-value 0.7061	Male	840 (87.87%)	116 (12.13%)
		Female	780 (88.44%)	102 (11.56%)
	Weight P-value 0.0008	Underweight (<2.5kg)	171 (84.65%)	31 (15.35%)
		Normal weight (2.5-4kg)	1396 (89.03%)	172 (10.97%)
		Overweight (>4kg)	43 (74.14%)	15 (25.86%)
	Gestational age P-value <0.0001	Preterm (<37w)	165 (79.33%)	43 (20.67%)
		Term (37-42w)	1451 (89.35%)	173 (10.65%)
		Post-term (>42w)	0 (0%)	2 (100%)

Table 3 shows the incidence of different types of CHD based on gender and different types of diabetes. Regarding the non-diabetic patients, more than half of them (66; 55%) were females, the remaining 54 (45%) were males, patent ductus arteriosus (PDA) had the highest incidence with 26 (31.7%) among males and 31 (32.97%) among females, patent foramen ovale (PFO) 24 (29.26%) for males and 31 (32.97%) for females, atrial septal defect (ASD) 23 (28.04%) among males and 23 (24.46%) among females, ventricular septal defect (VSD) seven (8.53%) among males and eight (8.51%) among females; one non-diabetic female had cardiomegaly. Only one male (1.2%) had dextro-cardia, among non-diabetic males 29 (53.7%) and among females 32 (48.48%) had multiple CHD. The distribution of different CHD types among type 1, type 2, and gestational diabetes regarding gender is shown in Table 3.

**Table 3:** the distribution of different CHD types based on DM type regarding the gender M - Male F - Female CHD - Congenital Heart Defect PDA - Patent Ductus Arteriosus PFO - Patent Foramen Ovale ASD - Atrial Septal Defect VSD - Ventricular Septal Defect PAA - Pulmonary Artery Aneurysm

Type of DM	CHD Incidence	PDA	PFO	ASD	VSD	PAA	Cardio- megaly	Dextro- cardia	Multiple
Non- diabetic	M 54 (45%)	26 (31.7%)	24 (29.26%)	23 (28.04%)	7 (8.53%)	1 (1.2%)	0 (0%)	1 (1.2%)	29 (53.7%)
	F 66 (55%)	31 (32.97%)	31 (32.97%)	23 (24.46%)	8 (8.51%)	0 (0%)	1 (1.06%)	0 (0%)	32 (48.48%)
Type 1 Diabetic	M 10 (41.67%)	5 (33.33%)	5 (33.33%)	3 (20%)	2 (13.33%)	0 (0%)	0 (0%)	0 (0%)	7 (70%)
	F 6 (31.58%)	2 (22.22%)	2 (22.22%)	4 (44.44%)	1 (11.11%)	0 (0%)	0 (0%)	0 (0%)	3 (50%)
Type 2 Diabetic	M 20 (29.41%)	7 (26.92%)	10 (38.46%)	6 (23.07%)	2 (7.69%)	0 (0%)	0 (0%)	1 (3.84%)	8 (40%)
	F 14 (24.56%)	8 (33.33%)	6 (25%)	8 (33.33%)	2 (8.33%)	0 (0%)	0 (0%)	0 (0%)	9 (64.28%)
Gestationa l Diabetes	M 32 (11.64%)	18 (34.61%)	17 (32.69%)	10 (19.23%)	4 (7.69%)	2 (3.84%)	0 (0%)	1 (1.92%)	16 (50%)
	F 16 (5.82%)	7 (31.81%)	7 (31.81%)	4 (18.18%)	4 (18.18%)	0 (0%)	0 (0%)	0 (0%)	6 (37.5%)

Table 4 shows the overall distribution of different CHD types based on DM. The incidences of PDA, PFO, ASD, VSD, cardiomegaly and dextro-cardia among the infants of non-diabetic patients were 57 (32.38%), 55 (31.25%), 46 (21.13%), 15 (8.52%), one (0.56%), one (0.56%), and one (0.56%) respectively and 61 (50.83%) had multiple CHD. Regarding type 1 diabetes mellitus, the incidences of different CHD among the infants were 16 (7.33%), seven (29.16%), seven (29.16%), seven (29.16%), three (12.5), 0 (0%), 0 (0%), and 0 (0%) respectively and 10 (62.5%) had multiple CHD. Concerning type 2 diabetes mellitus, the incidences of different CHD among the infants were 15 (30%), 16 (32%), 14 (28%), four (8%), 0 (0%), 0 (0%), and one (2%) respectively and 17 (50%) had multiple CHD. Finally, among gestational diabetic patients, the incidences of different CHD among the infants were 15 (30%), 16 (32%), 14 (28%), four (8%), 0 (0%), 0 (0%), and one (2%) respectively and 22 (45.83%) had multiple CHD. 

**Table 4:** the overall distribution of different CHD types based on diabetes mellitus (DM) CHD - Congenital Heart Defect PDA - Patent Ductus Arteriosus PFO - Patent Foramen Ovale ASD - Atrial Septal Defect VSD - Ventricular Septal Defect PAA - Pulmonary Artery Aneurysm

Type of DM	CHD Incidence	PDA	PFO	ASD	VSD	PAA	Cardio- megaly	Dextro- cardia	Multiple
Non- Diabetic	120 (55.04%)	57 (32.38%)	55 (31.25%)	46 (21.13%)	15 (8.52%)	1 (0.56%)	1 (0.56%)	1 (0.56%)	61 (50.83%)
Type 1 Diabetic	16 (7.33%)	7 (29.16%)	7 (29.16%)	7 (29.16%)	3 (12.5)	0 (0%)	0 (0%)	0 (0%)	10 (62.5%)
Type 2 Diabetic	34 (15.59%)	15 (30%)	16 (32%)	14 (28%)	4 (8%)	0 (0%)	0 (0%)	1 (2%)	17 (50%)
Gestational Diabetes	48 (22.01%)	25 (33.78%)	24 (32.43%)	14 (18.91%)	8 (10.81%)	2 (2.70%)	0 (0%)	1 (1.35%)	22 (45.83%)
Total incidence among diabetic	98 (100%)	47 (48%)	47 (48%)	35 (35.7%)	15 (15.3%)	3 (3.06%)	0 (0%)	2 (2.04%)	49 (50%

Table 5 compares the duration of diagnosis between different types of CHD in infants of diabetic and non-diabetic mothers. The diagnostic test used was echocardiography. Most of the cases (74.84%) were diagnosed within 24-72 hours post birth. Peak duration of diagnosis in our sample was within one to seven days post birth (86.33%). Fewer cases were diagnosed with duration more than one week (7.76%) and more than six weeks (0.62%). PDA, VSD and ASD were diagnosed earlier than the other types of CHD.

**Table 5:** Time to CHD diagnosis after birth CHD - Congenital Heart Defect PDA - Patent Ductus Arteriosus PFO - Patent Foramen Ovale ASD - Atrial Septal Defect VSD - Ventricular Septal Defect PAA - Pulmonary Artery Aneurysm

Duration	< 24 hours	24 - 72 hours	4-7 Days	> one week	> six weeks
						PDA	5 (4.85%)	80 (77.66%)	11 (10.67%)	7 (6.79%)	0 (0%)
PFO	1 (0.98%)	82 (80.39%)	13 (12.74%)	5 (4.90%)	1 (0.98%)
ASD	5 (6.17%)	57 (70.37%)	11 (13.58%)	8 (9.87%)	0 (0%)
VSD	4 (13.79%)	18 (62.06%)	2 (6.89%)	4 (13.79%)	1 (3.44%)
PAA	0 (0%)	3 (100%)	0 (0%)	0 (0%)	0 (0%)
Cardiomegaly	0 (0%)	1 (100%)	0 (0%)	0 (0%)	0 (0%)
Dextrocardia	2 (66.66%)	0 (0%)	0 (0%)	1 (33.33%)	0 (0%)
Total	17 (5.27%)	241 (74.84%)	37 (11.49%)	25 (7.76%)	2 (0.62%)

## Discussion

Maternal DM is a risk factor for adverse maternal and fetal outcomes, including anatomical malformations such as CHD [11]. The risk for CHD in offspring is present in mothers with all types of disease, such as type 1 or 2 diabetes mellitus existing before pregnancy, along with gestational diabetes mellitus developing during pregnancy [12,13]. Our study aimed to estimate infants with CHD whose mothers have diabetes at a tertiary hospital in NGHA in Saudi Arabia.

The incidence of CHD among infants of included mothers in this cohort study was 11.82%; similar studies were conducted by Alabdulgader et al. in the eastern province of Saudi Arabia [14] and Muhammad et al. in Peshawar-Pakistan [15] where they reported a lower incidence of CHD among infants, which was 10.7% and 9.3% respectively. However, Abu-Sulaiman and Subaih [16] reported a higher incidence of 15%. Differences in incidences of various CHD in all studies are due to different sample size selection and duration of study period. We had a very limited study period, while other studies have been conducted for five years [15].

Our results found that a total of 1838 infants were registered, out of which 677 (38.83%) were infants of diabetic mothers (IDMs). Our results did not match a local study conducted in Lahore by Aslam et al., who reported a total of 1530 newborns, out of which 84 (6%) were IDMs [17]. Among our diabetic mothers 98 (5.33%) of their infants had CHD; these results mismatched with Muhammad et al. who found a vast majority of IDMs (52.5%) had various congenital heart diseases [15]. They reported that their high incidence of congenital heart disease in IDMs could be because they had a small sample size for their hospital-based study.

We reported that 62 (63.3%) of the newborn infants of diabetic mothers were male and 36 (36.7%) were female, with an overall male to female ratio of 1.72:1; similar results were also found in the Muhammad et al. study where 66.30% were male and 33.7% were female with a ratio of 1.97:1 [15].

The incidence of congenital heart diseases in our study was slightly higher in pre-gestational infants (type 1 and type 2 diabetic mothers) than gestational ones, 50 (51%) and 48 (49%) respectively, while a similar study conducted by Behjati et al. reported that incidence of CHD was more frequent in infants of pre-gestational than gestational diabetic mothers, 49 (65%) and 36 (35%) respectively [18].

In our study, the most common echocardiographic findings in the IDMs were patent ductus arteriosus (PDA), and patent foramen ovale (PFO) were diagnosed in 48% of infants, atrial septal defect (ASD) in 35.7%, ventricular septal defect (VSD) in 15.3%, pulmonary artery aneurysm (PAA) in 3.06%, and dextro-cardia in 2.04%; these results were comparable with those of another Saudi study conducted by Abu-Sulaiman and Subaih who reported that regarding the CHD findings in IDMs, there were PDA in 70%, PFO in 68%, ASD in 5%, VSD in 4%, mitral valve prolapse in 2%, and pulmonary stenosis in 1% respectively [16]. In another study, researchers found that the most common echocardiographic findings in IDMs were asymmetrical septal hypertrophy in 80%, PFO in 37.5%, and PDA in 27.5% [19].

A recent Saudi study conducted in Jaddah by Hashim et al. [20] reported that 35 years old or younger mothers are more likely to have an infant with ASD, while babies of more than 35 years old mothers presented with VSD and patent ductus arteriosus. Our results found that the overall incidence of CHD was higher among mothers whose ages were between 30 and 34 years, at 14.52%. We found no significant correlation between mothers' age and the incidence of CHD among children.

Gestational age (GA) is a significant predictor of mortality in extremely preterm infants without congenital anomalies [21]. Bastek et al. reported that late preterm infants born at 34 to 36 weeks have a higher risk of death than term infants [22]. These results matched our results, which reported that all diabetic mothers with post-term gestational ages (two; 100%) had CHD. We also reported a significant correlation between gestational age and incidence of CHD.

Among the most consistently observed findings in newborns with CHD is reduced birth weight [23]. However, our results reported a significantly higher incidence of CHD among overweight infants.

## Conclusions

The incidence of CHD among infants of included mothers in this cohort study was 11.82%, with a higher prevalence of CHD among male infants (53.21%) than female ones (46.78%). The incidence of CHD was statistically higher among infants whose mothers had T1DM, followed by T2DM. However, in GDM and non-diabetic mothers, there was no statistical significance. The most common CHDs in IDMs were patent ductus arteriosus and patent foramen ovale. The incidence of CHD was higher among mothers whose ages were between 30 and 34 years. Gestational age is the most significant predictor for the incidence of CHD, where the post gestational age infants had a higher incidence of CHD. Unexpectedly, our results reported a significantly higher incidence of CHD among overweight infants.
